# Targeting AMPK for cancer prevention and treatment

**DOI:** 10.18632/oncotarget.3629

**Published:** 2015-03-20

**Authors:** Weidong Li, Shakir M. Saud, Matthew R. Young, Guohong Chen, Baojin Hua

**Affiliations:** ^1^ Department of Oncology, Guang'anmen Hospital, China Academy of Chinese Medical Sciences, Beijing, China; ^2^ Nutritional Science Research Group, Division of Cancer Prevention, National Cancer Institute, National Institutes of Health, Rockville, Maryland, USA; ^3^ Basic Research Laboratory, Center for Cancer Research, National Cancer Institute, National Institutes of Health, Frederick, Maryland, USA; ^4^ Department of Urinary Surgery, Guang'anmen Hospital, China Academy of Chinese Medical Sciences, Beijing, China

**Keywords:** AMP activated kinase, cancer, prevention, treatment

## Abstract

AMP-activated protein kinase (AMPK) is an important mediator in maintaining cellular energy homeostasis. AMPK is activated in response to a shortage of energy. Once activated, AMPK can promote ATP production and regulate metabolic energy. AMPK is a known target for treating metabolic syndrome and type-2 diabetes; however, recently AMPK is emerging as a possible metabolic tumor suppressor and target for cancer prevention and treatment. Recent epidemiological studies indicate that treatment with metformin, an AMPK activator reduces the incidence of cancer. In this article we review the role of AMPK in regulating inflammation, metabolism, and other regulatory processes with an emphasis on cancer, as well as, discuss the potential for targeting AMPK to treat various types of cancer. Activation of AMPK has been found to oppose tumor progression in several cancer types and offers a promising cancer therapy. This review evaluates the evidence linking AMPK with tumor suppressor function and analyzes the molecular mechanisms involved. AMPK activity opposes tumor development and progression in part by regulating inflammation and metabolism.

## INTRODUCTION

AMP-activated protein kinase (AMPK) is a highly conserved serine/threonine protein kinase consisting of a catalytic subunit (α) and two regulatory subunits (β and γ) and is expressed in a number of tissues, including liver and skeletal muscle [1]. The α-subunit of AMPK contains a conserved threonine residue (Thr172) by which phosphorylation by upstream protein kinases results in AMPK activation. Kinases that can activate AMPK include liver kinase B1 (LKB1), calcium/calmodulin-dependent protein kinase (CaMKK) [2] and transforming growth factor β (TGF-β)-activated kinase (TAK1) [3]. Individuals with Peutz-Jeghers syndrome that have germline mutations in LKB1 have a higher prevalence of cancer [2]. AMPK can also be activated by extracellular changes, such as, depletion of ATP, low glucose, and changes of NADPH levels [4]. Administration of drugs and certain naturally occurring compounds can also activate AMPK. Metformin and some non-steroidal anti-inflammatory drugs (NSAIDs) can activate AMPK [5, 6]. Polyphenols (resveratrol) [7-9], Flavonoids (quercetin) [10], and Chinese herbal compounds (berberine) [11-13] have also been shown to activate AMPK. AMPK activation by NSAID's and other anti-inflammatory agents has implicated a potential role of AMPK during inflammation. Once activated AMPK can influence many effectors proteins involved in various regulatory processes that contribute to the pathogenesis of cancer. In relation to cancer metabolism mammalian target of Rapamycin (mTOR) is an important AMPK target with many efforts being made to target it in the clinic [14]. AMPK can also regulate p53 [15] and modulate the activity of transcription factors and co-regulators that control the cell cycle [16-18]. The current evidence suggests that AMPK can act as a tumor suppressor by modulating inflammation, opposing metabolic changes that occur during tumorigenesis and directly inducing cell-cycle arrest [19]. This review will provide an overview of AMPK role as a tumor suppressor and its therapeutic potential for the prevention and treatment of cancer.

### AMPK: state of the art

AMPK was first described because of its role in lipid metabolism and regulating cholesterol and fatty acid levels [20, 21]. Since then the role of AMPK in regulating cellular energy homeostasis places this enzyme as a major regulator of energy metabolism. Generally speaking AMPK is activated when cellular energy is altered. A number of stresses can activate AMPK, including glucose deprivation, ischemia, hypoxia and oxidative stress [22]. Once activated AMPK phosphorylates numerous metabolic enzymes acutely inhibiting pathways that consume ATP and activating pathways that generate ATP, such as, glucose uptake and fatty acid oxidation [23, 24]. AMPK's role in regulating metabolism is well understood; predominately studied in the context of type-2 diabetes and metabolic syndrome. Li et al. 2013 describes a series of experiments elucidating the role of AMPK activation in treating diabetes. Treating primary hepatocytes with an AMPK activator known as C24 resulted in an inhibition of glucose production by down regulation of phosphoenol pyruvate carboxykinase (PEPCK) and glucose-6-phosphatase (G6Pase), known genes involved in gluconeogenesis that correlated with a decrease in triglyceride and cholesterol levels. When C24 was administered to diabetic mice (db/db) it alleviated symptoms associated with diabetes, lowering blood glucose, cholesterol and circulating free fatty acids [25]. AMPK activation can prevent atherosclerosis and reperfusion injury of the heart in experimental animals [26, 27]. Conversely, dysregulation of AMPK activation has been found to be associated with the risk of developing insulin resistance (IR) and metabolic syndrome–associated diseases in both experimental animal models and in clinical studies [28]. AMPK inhibits essentially all anabolic pathways that promote cell growth including fatty acids, phospholipids, protein and ribosomal RNA synthesis [1, 24]; thus, in cancer where the energy demands of the cell are elevated due to rapid growth and division AMPK activators may be a suitable therapeutic intervention for treating cancer.

### Relevance of AMPK to cancer

Deregulating cellular energetics is a core hallmark of cancer [29-31]. AMPK activation may act as a metabolic tumor suppressor by regulating energy levels, enforcing metabolic checkpoints and inhibiting cell growth. There is a vast literature demonstrating the tumor suppressor function of AMPK in lung, colorectal, and liver cancer with a growing literature in other cancers, such as, prostate and melanoma.

#### AMPK and lung cancer

Non-small-cell lung cancer (NSCLC) is the most common lung cancer type, accounting for 75-80 percent of all lung cancer cases [32]. It is estimated that 30-50 percent of NSCLC have mutations in the gene coding for LKB1 [33, 34]. It is hypothesized that mutations in LKB1 result in unsuppressed cell proliferation due to the inability to activate AMPK in response to the tumor [35]. William et al. isolated tumors from patients with NSCLC and found that AMPK activation correlated with a better prognosis and a significant increase in overall survival [32]. Furthermore, AMPK activity was significantly higher in lung tumors obtained from never smokers than in smokers. Similar results were seen in a study investigating the correlation of LKB1 mutations with several clinicopathological characteristics of 155 patients with lung adenocarcinoma that found LKB1 mutations associated with smokers and not nonsmokers [36]. Specific AMPK activators may be useful in treating NSCLC patients with LKB1 mutations and history of smoking.

#### AMPK and colorectal cancer

Colorectal cancer (CRC) is the third leading cause of cancer-related mortality in the U.S. with no effective therapy for advanced colorectal carcinoma [37]. Colorectal cancer has a strong inflammatory and metabolic component suggesting AMPK activation may be useful in colorectal cancer management. The direct relationship between AMPK activation and colon cancer survival has yet to be established. A recent study with several hundred colorectal cancer samples was unable to find a correlation between overall survival and AMPK activation. However, the study was able to demonstrate that AMP activation correlated with mitogen activated protein kinase (MAPK) activation and within that particular subset, AMPK activation was associated with a significant decrease in cancer-specific mortality [38]. Several reports have demonstrated that activating AMPK by 5-aminoimidazole-4-carboxamide-ribonucleoside (AICAR) or phenformin in human cancer cells results in apoptosis by several mechanisms, including modulating the MAPK pathway [39, 40]. Taken together, these findings suggest AMPK activation may be beneficial in regulating cell survival in colorectal cancer tumor types.

#### AMPK and liver cancer

The most common type of liver cancer is hepatocellular carcinoma (HCC), which is the fourth most commonly diagnosed cancer in the U.S. [41]. The link between AMPK and normal liver function is very evident due to the important role of the liver in regulating fatty acid oxidation and lipid metabolism. Diseases of the liver including HCC are often associated with metabolic disorder [42]. Loss of LKB1 and AMPK expression correlates with a poor prognosis. In patient samples collected prior to hepatectomy, low LKB1 expression correlated with a greater degree of tumor severity and significantly shorter disease-free survival [43]. Similarly, decreased AMPK activity in patient tumors correlated with an aggressive clinical phenotype and poor prognosis [44]. It appears that the LKB1-AMPK pathway directly influences proliferation of HCC cells [45]. *In vitro* knockdown of AMPK in HCC cells resulted in greater tumorigenicity when implanted into nude mice [11]. While the mechanisms are still under investigation, AMPK activation appears to attenuate HCC by inducing cell senescence [46] and autophagy [47].

#### Role of AMPK in other cancers

More recently, pre-clinical studies have shown AMPK having some involvement in others cancers, including melanoma [11, 48-52], breast cancer [53-56], prostate cancer [57, 58], ovarian cancer [59, 60] and leukemia's [61]. In melanoma, AMPK was found to be an important regulator for the maintenance of MITF (Microphthalmia-associated transcription factor), a protein important for normal melanocyte development and differentiation that is associated with melanoma progression [50]. AMPK activation has also been shown to inhibit the metastatic potential of melanoma cells through a reduction in the activity of the ERK signaling pathway and COX-2 protein levels [11] and by inducing autophagic cell death and apoptosis through AMPK/JNK signaling [51]. In primary breast cancer, AMPK activity is diminished in an estimated 90% of cases [62]. Fox et al. demonstrated that reintroducing AMPKα2 suppressed the growth of MCF-7 breast cancer cells [55]; whereas, in a different study overexpressing a constitutively active form of AMPK reduced cell death induced by low glucose [56]. In Chronic Myeloid Leukemia (CML), BCL-ABL transformed cells exhibit overly expressed mTOR activity by which activating AMPK may provide therapeutic advantages [61]. Based on the evidence, AMPK does exhibit tumor suppressor-like activation in certain primary cancers; however, there are some tumors and cellular contexts in which the proposed role may not be applicable and more investigation is warranted.

### Cancer Related Targets of AMPK

#### mTOR

Mammalian target of Rapamycin (mTOR) is a serine/threonine protein kinase that regulates cell growth, cell proliferation, cell motility, cell survival, protein synthesis, and transcription [63]. mTOR forms two functionally distinct complexes, mTORC1 (mTOR complex 1) and mTORC2 (mTOR complex 2). AMPK inhibits mTOR through the phosphorylation of tuberous sclerosis complex protein-2 (TSC2), which converts the small G protein Rheb to its inactive GDP form and the phosphorylation of Regulatory associated protein of mTOR(Raptor) proteins that ultimately leads to inhibition of downstream targets p70s6kinase and activation of 4EBP, which are directly involved in translation and protein synthesis [64]. The activated TSC1/TSC2 complex regulates the activity of mTORC1 and raptor, which controls cell growth mainly through the regulation of protein translation. The AMPK-mTOR axis can also regulate autophagy, a catabolic degradation process within the cell. Activation of autophagy can lead to tumor growth by maintaining energy production and offers another therapeutic advantage of AMPK activation [65]. Inhibition of mTORC1 is sufficient to induce autophagy in the presence of nutrients in yeast or mammalian cells, establishing mTORC1 as a conserved and critical repressor of autophagy [66]. AMPK regulates several metabolic processes and activates the TSC to repress mTORC1 under conditions of energy stress. AMPK also directly regulates autophagy by phosphorylating and activating UNC-51-like kinase1 (ULK1) at Ser317, a key initiator of autophagy that is negatively regulated by the mTOR kinase [67-69]. Concomitant with suppression of mTORC1 autophagy is induced accompanied by loss of cell viability [70].

#### COX-2

Cyclooxygenase-2 (COX-2) is a known pro-inflammatory enzyme that has been shown to be up regulated in a number of cancers and correlates with tumorigenesis [71, 72]. COX-2 is responsible for the formation of important pro-inflammatory mediators, including prostanoids, which have been shown to promote tumor growth in a variety of cancers [73, 74]. While there is limited evidence demonstrating a direct link, several studies were able to show a correlation between AMPK activation by numerous agents and COX-2 inhibition in colorectal cancer cell lines and xenografts [75, 76], as well as, leukemic [77] and melanoma cell lines [11]. While there are several COX-2 inhibitors available in the clinic the use of these selective COX-2 inhibitors are limited by their side effects [78, 79]. Thus, activating AMPK may provide a novel approach to inhibiting COX-2 at sites of inflammation, such as, within the tumor microenvironment.

#### p53

p53 is a tumor suppressor that plays an important role in preventing tumor development by responding to a number of cellular stresses, including DNA damage, oncogene activation and hypoxia and inducing cell cycle arrest or senescence [80, 81]. It is estimated that p53 is inactivated in approximately 50 percent of human cancers [82]. Many kinases stabilize p53 by phosphorylation under conditions of metabolic stress. AMPK can directly phosphorylate p53 leading to its stabilization and transcriptional activity [15] and promote p53 gene expression [83]. In fact, in a study utilizing murine embryonic fibroblast the absence of AMPKα2 tumors exhibited decreased p53 expression and enhanced cell growth and transformation [84]. Under low-energy status AMPK activation maintains energy homeostasis for normal cellular events and also induces p53 to restrict cell growth rates to save more energy to limit induction of potential damage in energy-rich environments. A recent study revealed a prosurvival role for p53 in cells metabolically impaired by glucose limitation [15]. Activation of p53 allows cells to respond to glucose deprivation by arresting their proliferation until glucose is restored. The ability of glucose deprivation to induce p53 was found to be AMPK dependent. Although loss of p53 confers a selective growth advantage to cancer cells, loss of p53 impairs the ability of cancer cells to respond to metabolic changes induced by metformin or AICAR and to survive under conditions of nutrient deprivation [85]. Metformin can also inhibit cancer cell proliferation in p53 deficient cells [86].

#### Acetyl-CoA carboxylase (ACC)

Acetyl-CoA carboxylase (ACC) is a well-established downstream target of AMPK involved in lipid metabolism [87]. ACC catalyzes acetyl-CoA carboxylation to produce malonyl-CoA a substrate for fatty acid biosynthesis and inhibitor of fatty acid uptake [88-90]. In several cancers, tumor progression is accompanied by marked changes in the expression of enzymes involved in fatty acid homeostasis, including ACC. In several cases, cancer cell proliferation and survival are dependent on ACC activity and inhibiting ACC results in apoptosis [91, 92].

#### Akt

The signaling relationship between Akt and AMPK is quite complex. On one hand, Akt has been shown to be a negative regulator of AMPK and upstream positive regulator of mTOR. Both pathways involve direct phosphorylation of TSC2, a negative regulator of mTOR. AMPK activates TSC2 and Akt inhibits TSC2 both leading to mTOR activation and subsequent increase in protein synthesis and other cellular processes. The negative regulation of AMPK by Akt involves the regulation of cellular ATP levels. In cells with activated Akt there is depleted ATP and decline in AMPK activity [93]. On the other hand other studies have shown that activated AMPK can also induce Akt phosphorylation and activation [94, 95]. To further complicate matters in some circumstances activating AMPK can inhibit Akt signaling. When cancer cells are treated with AICAR, a widely used AMP-Kinase activator, it resulted in S-phase growth arrest in an LKB1-independent fashion by inducing p53 and inhibiting Akt phosphorylation [96]. Thus, the cross talk between AMPK and Akt is bidirectional, yet the functional consequence in terms of tumor progression is unclear. Choudhury et al. using various prostate cancer cell lines was able to illustrate this bidirectional feedback mechanism. Treating androgen-independent PC3 and PC3M cells with AICAR, an AMPK activator depending on dose or time either activated or inhibited Akt; however, in both cases mTOR activity and tumor progression was inhibited, which was found to be independent of PI3K-Akt signaling [97]. Taken together, depending on the circumstances activating AMPK may inhibit or promote Akt signaling; however, the phenotypic consequences that follow may be dependent on the tumor and cellular context. Further investigation and careful consideration of the feedback mechanism between AMPK and PI3K will be needed when [94, 95] targeting AMPK for cancer treatment.

### How AMPK modulates inflammation

The link between inflammation and cancer has been well established; however, the mechanism by which AMPK control on metabolism can directly influence inflammation and tumorigenesis is still unclear. The most intuitive relationship is that immune cells like most cells have energy demands, especially during inflammatory pathologies, including diabetes and cancer. It is now becoming evident that unstimulated or naive immune cells, including dendritic cells, neutrophils, macrophages and T-cells utilize mainly oxidative metabolism, including fatty acid oxidation to generate ATP. However, when activated by pro-inflammatory cytokines, binding of ligands to TLRs (Toll-like receptors), or antigen presentation the immune cells switch to the use of aerobic glycolysis instead [98]. Since AMPK is important regulator of these metabolic processes it is possible that AMP-activating drugs can modulate inflammation. Indeed, Carroll, et al. found that AMPKα1-deficient macrophages and DCs exhibit heightened inflammatory function and an enhanced capacity for antigen presentation favoring the promotion of Th1 and Th17 responses. Th1 and Th17 responses play a complex and controversial role in tumor immunity either promoting or suppressing tumor growth. A second study, revealed that Macrophages and DCs generated from AMPKα1-deficient mice produced higher levels of pro-inflammatory cytokines and decreased production of the anti-inflammatory cytokine IL-10 when activated compared with WT cells [99]. Pro-inflammatory cytokines have been correlated with tumor progression in various cancers. Lung cancer is highly inflammatory and managing the inflammation has been shown to inhibit tumorigenesis. Hoogendijk, et al. found that activation of AMPK *in vitro* reduced cytokine production in the alveolar macrophage cell line MH-S. *In vivo*, AMPK activation inhibited lung inflammation by reducing Lipoteichoic acid-induced neutrophil influx and by altering cytokine/chemokine levels in the bronchoalveolar space [100]. Inflammation is a one of the classic hallmarks of cancer and targeting AMPK is this context may beneficial for cancer treatment.

### Targeting AMPK for Cancer Prevention and Treatment

#### Metformin

Metformin is a drug used to decrease hyperglycemia in patients with type-2 diabetes, in part by activating AMPK and is currently under investigation as a potential treatment for several types of cancer [101, 102]. Epidemiological and clinical data suggest a benefit of metformin treatment in preventing certain cancers [103-106]; however, the molecular mechanisms are incompletely understood. Recent preclinical studies were able to demonstrate metformin efficacy in various cancer types. In NSCLC cell lines metformin inhibited proliferation and made the cells more sensitive to growth ionizing radiation [107] and *in vivo* metformin was found to prevent tobacco carcinogen-induced lung tumorigenesis [108]. Similarly, metformin significantly increased the radiosensitivity of luminal-type MCF-7 breast cancer cells [101]. Metformin abolished the self-renewal capabilities and induced apoptosis in HCC cell lines [109] and low dose metformin inhibited adipocyte-dependent proliferation in ID8 mouse ovarian cancer cell lines [102]. Metformin was also shown to significantly reduce aberrant crypt foci, the precursors to colon polyps with a modest reduction in polyp formation in animals treated with chemical carcinogen Azoxymethane [110]. In melanoma, metformin inhibits invasion and metastasis development through AMPK/p53 axis activation [49]. While there remains uncertainty regarding whether or not AMPK activation is required for metformin benefits [111] it still remains clear that metformin can activate AMPK and may be useful in the prevention and treatment of cancer.

#### Non-steroidal anti-inflammatory drugs (NSAIDs)

Inflammation has been shown to play a critical role in tumor initiation, progression, and metastasis [112]. Aspirin, a non-steroidal anti-inflammatory drug (NSAID) has been shown to correlate with a decreased risk of developing cancer, particularly preventing colorectal cancer (CRC) [113, 114]. The majority of the studies are observational in nature and the mechanisms are currently being investigated; however, it is believed that aspirin may prevent CRC by inhibiting COX-2 [115]. This ultimately led to the development of a new class of NSAIDs that are more selective for COX-2 inhibition for use as chemopreventive agents. Interestingly, aspirin and other NSAIDs have also been shown to activate AMPK. When colorectal cancer cells were treated with aspirin, there was a significant increase in AMPK activation and inhibition of downstream mTOR signaling [116]. Aspirin use has also been shown to correlate with the prevention of several other cancers including lung, liver and ovarian; however, the molecular mechanisms involving its prevention remains enigmatic. It is likely that once aspirin or other NSAIDs activate AMPK there is regulation of other inflammatory regulatory pathways. For example in the lung, AMPK activation can alleviate inflammation that results from injury or infection by directly modulating the activity of macrophages, neutrophils and T cells [99, 100, 117]. Leukocytes and their cytokines play important regulatory roles in all aspects of tumor development; therefore the anti-inflammation drugs targeting AMPK activation may play a more meaningful role for the treatment of cancer-associated inflammation.

#### Natural products

**Figure 1:**
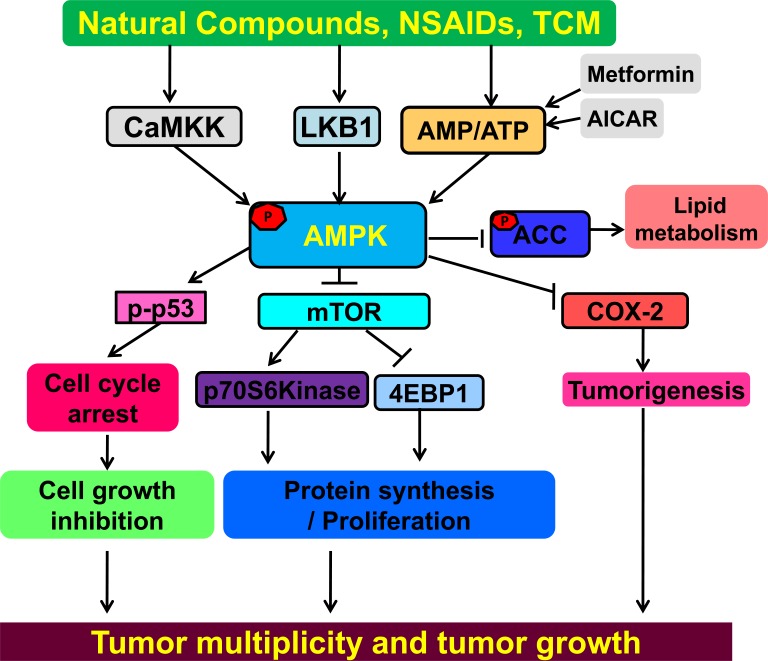
AMPK is a tumor suppressor for cancer prevention and treatment NSAIDs, Natural products, TCM and metformin can all activate AMPK. AMPK negatively regulates the mTOR signal pathway, resulting in inhibition of cancer proliferation and growth. Activated AMPK negatively regulates COX-2, a pro-inflammatory enzyme associated with tumorigenesis. AMPK can induce phosphorylation of tumor suppressor p53, resulting in cell cycle arrest. Activation of AMPK can also induce phosphorylation of ACC influencing lipid metabolism. Interactions leading to activation of molecular targets are indicated by arrows; those inhibited are indicated by a bar. Activation of AMPK can modulate multiple pathways leading to anticancer activities. TCM=Traditional Chinese Medicine; NSAIDs=Non-steroidal anti-inflammatory drugs.

The use of natural products as chemopreventive agents has increased worldwide because of their potential low toxicity and effectiveness [118]. Observational studies have suggested the efficacy of some natural products in preventing the development of cancer; however, clinical trials have yet to be conducted in most cases to support this notion. In preclinical studies, several classes of natural products have been shown to target many mediators that play important roles in cancer, including AMPK (Figure 1 and Table 1). Flavones, such as wogonin [119], tanshinone IIA [120], quercetin [121, 122] and cryptotanshinone [123] induce AMPK activation inhibiting proliferation and inducing apoptosis in various types of cancer cells. Polyphenols are also an abundant source of AMPK activators. Resveratrol induces autophagy in chronic myelogenous leukemia cells by regulating the AMPK/mTOR pathway [124]. In colorectal cancer cells, magnolol [125], epigallocatechin-3-gallate (EGCG) [126], and widdrol [127] could induce apoptosis, inhibit migration, and prevent invasion by AMPK-dependent mechanisms. In breast cancer cells, nordihydroguaiaretic acid inhibits mTORC1 activity through disruption of mTOR-Raptor complex and AMPK activation [128], while demethoxycurcumin, a potent AMPK activator has broad spectrum anticancer activity in triple-negative breast cancer cells [129] with the parent compound curcumin exerting similar effects in ovarian cancer cells [130] and in colorectal cancer cells by AMPK-p53 activation [131, 132]. Antroquinonol, extracted from *antrodiacamphorate* displays anti-cancer activity against HCC cells through an AMPK-dependent manner [133] and honokiol, has been shown to inhibit proliferation in several cancer cell types, also through AMPK dependent mechanisms [134]. Berberine has been shown to inhibit colon tumor formation in AOM/DSS mouse model through activation of AMPK [135]. Berberine [11]or Ginsenoside 20-O-b-D-Glucopyranosyl-20(S)-Protopanaxadiol [48] inhibit melanoma cell growth and invasion through the activation of AMPK *in vitro* [11, 48-52]. Thus, these agents may serve as AMPK activators and provide a crucial link between natural products and the prevention and treatment of cancer.

**Table 1:** Natural products extracted from herbal medicines that can activate AMPK to inhibit the growth of multiple cancer types

Natural products	Effect of AMPK activation	Cancer type	References
Berberine	Inhibition metastasis by AMPK/ERK	Melanoma	[11]
Ginsenoside 20-O-b-D-Glucopyranosyl-20(S)-Protopanaxadiol	Induces autophagic cell death by AMPK/JNK	Melanoma	[48]
Wogonin	Inhibition translation by AMPK/mTOR/4EBP1	Glioblastoma	[119]
			
Tanshinone IIA	Induces autophagic cell death by AMPK/mTOR/p70S6kinase	Leukemia	[120]
Quercetin	Growth inhibition through AMPK/COX-2	Breast and colon cancer	[121, 122]
Cryptotanshinone	Induce autophagic cell death by AMPK/mTOR	Hepatoma and colon cancer	[123]
Resveratrol	Triggered autophagic cell death via AMPK/mTORC2/p62	Leukemia	[124]
Magnolol	Induce apoptosis of colon cancer by AMPK/p53	Colon cancer	[125]
Epigallocatechin-3-gallate	Suppress colon cancer proliferation by ROS/AMPK/COX-2	Colon cancer	[126]
Widdrol	Induction of apoptosis via AMPK	Colon cancer	[127]
Nordihydroguaiaretic acid	Inhibition breast cancer growth by AMPK/mTORC1	Breast cancer	[128]
Demethoxycurcumin	Inhibition breast cell growth by AMPK/mTORC1	Breast cancer	[129]
Curcumin	Suppress proliferation by AMPK/p53	Ovarian cancer	[130, 131]
Antroquinonol	Anticancer by AMPK/mTOR/p70s6kinase and 4EBP1	Hepatocellular carcinoma	[133]
Honokiol	Inhibition invasion and migration of breast cancer by LKB1/AMPK/mTOR	Breast cancer	[134]
Berberine	Inhibition growth by AMPK/mTOR and AMPK/COX-2	Colon cancer	[135]

#### AICAR

5-aminoimidazole-4-carboxamide ribonucleotide (AICAR) is the pharmacologic activator of AMPK. AICAR is transported in the cell by the adenosine transporter and is metabolized to an AMP analog, ZMP, which in turn binds to the γ-subunit of AMPK, thus enabling the activating phosphorylation of AMPK by LKB1 on Thr172 [136]. AICAR mediated AMPK activation has been reported to inhibit cell proliferation and cell cycle progression via inhibition of the PI3K/Akt pathway and the cell cycle regulatory proteins p21, p27 and p53 [96]. AICAR-mediated AMPK activation was found to be a proficient cytotoxic agent in Acute lymphoblastic leukemia (ALL) cells and the mechanism of its anti-proliferative and apoptotic effect appear to be mediated via activation of p38-MAPK pathway, increased expression of cell cycle inhibitory proteins p27 and p53, and downstream effects on the mTOR pathway, hence exhibiting therapeutic potential as a molecular target for the treatment of childhood ALL [137]. In human colorectal cancer cells AICAR through AMPK signaling pathway sensitizes death receptor-mediated cytotoxicity [138]. These findings suggest that AICAR can be used alone or combined with chemotherapies for cancer treatment.

#### Traditional Chinese medicine

The use of traditional Chinese medicine (TCM) for cancer prevention and treatment has stimulated much interest in recent years [139]. TCM integrates a wide range of herbal medicines often used in combinations of more than four single herbal medicines. The use of TCM in treating metabolic disorders has been well established. There are at least twenty medicinal herbs in TCM used for the treatment of metabolic disorders that involve some regulation of mitochondrial function, stimulation of glycolysis and AMPK activation [140]. Jiaotaiwan (JTW), composed of *Coptischinensis* (CC) is one of the most widely used agents for treating diabetes and the mechanism of action involves AMPK activation in the liver and increased glucose uptake [141]. Hugan Qingzhi tablet (HQT), a lipid-lowering traditional Chinese medicine formula has been shown to exert a preventive effect against hepatic steatosis and its mechanism of action may involve activation of AMPK and PPARα pathways [142]. Activation of AMPK by TCM agents has produced similar results seen with metformin. Thus, many herbal medications used in traditional Chinese medicine to treat chronic inflammation and diabetes, are potential candidates for targeting AMPK for prevention and treatment of cancer.

### The potential drawbacks of activation of AMPK

While targeting AMPK has become an attractive target for cancer therapy there are cases in which AMPK activation may promote cancer. In the case of mTOR, isoform specificity is particularly important. Most cancers have activation in mTOR complex 1 (mTORC1), which regulates growth through effectors, such as 4EBP1 and S6K1, as discussed above. Thus, inhibiting mTORC1 will prevent cell protein synthesis and proliferation; however, inhibiting mTORC1 without inhibiting mTOR complex 2 (mTORC2) can activate PI3K-Akt signaling pathway and promote tumor survival as previously mentioned. Activation of AMPK in most cases results in mTORC1 inhibition; however, the effects on mTORC2 and Akt activation are incompletely understood. If a particular AMPK activator is to be used solely to regulate mTOR its success would depend on its capacity to inhibit both complexes. AMPK agonist in recent studies has shown to possess both tumor suppressing and tumor promoting abilities, which may result from mechanisms relating to feedback regulation [143, 144]. In the case of prostate cancer AMPK activation may be associated with poor prognosis. Ca2+/CaM-dependent protein kinase kinase β (CaMKKβ) is elevated and correlated with prostate cancer cell migration and proliferation. Recall that CaMKKβ is an upstream activator of AMPK. Inhibition of CaMKII activity by synthetic agents has been shown to suppress prostate cancer cell growth [145]. CaMKKβ-induced prostate cancer cell migration requires AMPK activation [58] and blocking CaMKK/AMPK pathway results to the inhibition of prostate cancer cell growth. It is likely that in the case of cancer treatment AMPK activation may be cell type and context dependent and will be one of the most difficult conundrums to address in future studies.

### Future perspectives

A considerable amount of evidence supports the notion that AMPK activation may act as a metabolic tumor suppressor. AMPK activation, whether direct or indirect has been shown to alleviate symptoms associated with type-2 diabetes and metabolic syndrome and has been a well-established therapeutic for these particular disorders. Epidemiological studies suggest that patients prescribed metformin, a drug commonly used to treat type-2 diabetes have a lower risk of developing cancer; conversely, patients with diabetes have higher incidences of cancer. It is hypothesized that AMPK activation can oppose tumor development by reprogramming cellular metabolism targeting one of the fundamental requirements necessary for cancer to develop and progress. In several cancers loss of AMPK signaling is associated with a worse clinical outcome in lung, colon and liver cancer. Future studies would have to further delineate whether or not loss of AMPK activity increases the susceptibility to cancer, as in the case of loss of LKB1. It is plausible considering the well-established cancer-related targets that are known to be regulated by AMPK, including, p53, COX-2, ACC, and mTOR (Figure 1).

In our opinion, the more exciting outcomes for activating AMPK for chemoprevention will come from the area least studied in relation to AMPK and that is inflammation. Considering that AMPK is activated by NSAIDs and agents that are traditionally seen as anti-inflammatory raises an important question as to whether the chemopreventive activity of AMPK activation is related to its ability to modulate inflammation. Traditional Chinese medicine (TCM) has been used for hundreds of years to prevent and treat many maladies, including cancer. The use of TCM has been linked to low incidence of certain cancers. Interestingly, many of the herbal remedies historically used in TCM have been used to alleviate inflammation, and recent reports contribute this to the anticancer activities seen with these compounds. It is tempting to speculate that AMPK-mediated anticancer activities may be related to inflammation and contribute to the effectiveness of TCM and potentially other AMPK activators. Currently, most of the research involving AMPK involves metabolism and only recently has work begun to unravel the direct role of AMPK in inflammatory processes and how it may relate to the anti-cancer activities seen by metformin, NSAIDs, TCM and other AMPK activators. Furthermore, there are all the other indirect effects of AMPK activation that still have to be investigated. Therefore, additional studies are necessary before AMPK activators can be used for clinical use for cancer prevention and treatment.

## Abbreviations

AMPK: AMP-activated protein kinase
CaMKK: calcium/calmodulin-dependent protein kinase
NSAIDs: non-steroidal anti-inflammatory drugs
mTOR: mammalian target of rapamycin
4EBP1: eukaryotic translation initiation factor 4E binding protein 1
NSCLC: Non-small-cell lung cancer
CRC: Colorectal cancer
HCC: hepatocellular carcinoma
ACC: acetyl-CoA carboxylase
LKB1: Liver Kinase B1
COX-2: Cyclooxygenase 2

## References

[1] Hardie DG (2007). AMP-activated/SNF1 protein kinases: conserved guardians of cellular energy. Nature reviews Molecular cell biology.

[2] Shaw RJ, Kosmatka M, Bardeesy N, Hurley RL, Witters LA, DePinho RA, Cantley LC (2004). The tumor suppressor LKB1 kinase directly activates AMP-activated kinase and regulates apoptosis in response to energy stress. Proceedings of the National Academy of Sciences of the United States of America.

[3] Chen Z, Shen X, Shen F, Zhong W, Wu H, Liu S, Lai J (2013). TAK1 activates AMPK-dependent cell death pathway in hydrogen peroxide-treated cardiomyocytes, inhibited by heat shock protein-70. Molecular and cellular biochemistry.

[4] Jeon SM, Chandel NS, Hay N (2012). AMPK regulates NADPH homeostasis to promote tumour cell survival during energy stress. Nature.

[5] Yao F, Ji GY, Zhang L (2012). [AMPK: a novel target controlling inflammation]. Sheng li xue bao: [Acta physiologica Sinica].

[6] Yue W, Yang CS, DiPaola RS, Tan XL (2014). Repurposing of metformin and aspirin by targeting AMPK-mTOR and inflammation for pancreatic cancer prevention and treatment. Cancer prevention research.

[7] Hwang JT, Kwak DW, Lin SK, Kim HM, Kim YM, Park OJ (2007). Resveratrol induces apoptosis in chemoresistant cancer cells via modulation of AMPK signaling pathway. Annals of the New York Academy of Sciences.

[8] Lin JN, Lin VC, Rau KM, Shieh PC, Kuo DH, Shieh JC, Chen WJ, Tsai SC, Way TD (2010). Resveratrol modulates tumor cell proliferation and protein translation via SIRT1-dependent AMPK activation. Journal of agricultural and food chemistry.

[9] Rashid A, Liu C, Sanli T, Tsiani E, Singh G, Bristow RG, Dayes I, Lukka H, Wright J, Tsakiridis T (2011). Resveratrol enhances prostate cancer cell response to ionizing radiation. Modulation of the AMPK, Akt and mTOR pathways. Radiation oncology.

[10] Shen Y, Croft KD, Hodgson JM, Kyle R, Lee IL, Wang Y, Stocker R, Ward NC (2012). Quercetin and its metabolites improve vessel function by inducing eNOS activity via phosphorylation of AMPK. Biochemical pharmacology.

[11] Kim HS, Kim MJ, Kim EJ, Yang Y, Lee MS, Lim JS (2012). Berberine-induced AMPK activation inhibits the metastatic potential of melanoma cells via reduction of ERK activity and COX-2 protein expression. Biochemical pharmacology.

[12] Li Y, Wang P, Zhuang Y, Lin H, Li Y, Liu L, Meng Q, Cui T, Liu J, Li Z (2011). Activation of AMPK by berberine promotes adiponectin multimerization in 3T3-L1 adipocytes. FEBS letters.

[13] Jeong HW, Hsu KC, Lee JW, Ham M, Huh JY, Shin HJ, Kim WS, Kim JB (2009). Berberine suppresses proinflammatory responses through AMPK activation in macrophages. American journal of physiology Endocrinology and metabolism.

[14] Wullschleger S, Loewith R, Hall MN (2006). TOR signaling in growth and metabolism. Cell.

[15] Jones RG, Plas DR, Kubek S, Buzzai M, Mu J, Xu Y, Birnbaum MJ, Thompson CB (2005). AMP-activated protein kinase induces a p53-dependent metabolic checkpoint. Molecular cell.

[16] Leclerc I, Lenzner C, Gourdon L, Vaulont S, Kahn A, Viollet B (2001). Hepatocyte nuclear factor-4alpha involved in type 1 maturity-onset diabetes of the young is a novel target of AMP-activated protein kinase. Diabetes.

[17] Kawaguchi T, Osatomi K, Yamashita H, Kabashima T, Uyeda K (2002). Mechanism for fatty acid “sparing” effect on glucose-induced transcription: regulation of carbohydrate-responsive element-binding protein by AMP-activated protein kinase. The Journal of biological chemistry.

[18] Liang J, Shao SH, Xu ZX, Hennessy B, Ding Z, Larrea M, Kondo S, Dumont DJ, Gutterman JU, Walker CL, Slingerland JM, Mills GB (2007). The energy sensing LKB1-AMPK pathway regulates p27(kip1) phosphorylation mediating the decision to enter autophagy or apoptosis. Nature cell biology.

[19] O'Neill LA, Hardie DG (2013). Metabolism of inflammation limited by AMPK and pseudo-starvation. Nature.

[20] Habegger KM, Hoffman NJ, Ridenour CM, Brozinick JT, Elmendorf JS (2012). AMPK enhances insulin-stimulated GLUT4 regulation via lowering membrane cholesterol. Endocrinology.

[21] Rodrigue-Way A, Keil S, Caron V, Bilodeau S, Levy E, Mitchell GA, Tremblay A (2013). Scavenger Receptor CD36 Mediates Inhibition of Cholesterol Synthesis via Activation of the LKB1-AMPK Pathway and Insig1/Insig2 Expression in Hepatocytes. Canadian journal of diabetes.

[22] Mihaylova MM, Shaw RJ (2011). The AMPK signalling pathway coordinates cell growth, autophagy and metabolism. Nature cell biology.

[23] Viollet B, Horman S, Leclerc J, Lantier L, Foretz M, Billaud M, Giri S, Andreelli F (2010). AMPK inhibition in health and disease. Critical reviews in biochemistry and molecular biology.

[24] Hardie DG, Ross FA, Hawley SA (2012). AMP-activated protein kinase: a target for drugs both ancient and modern. Chemistry & biology.

[25] Li YY, Yu LF, Zhang LN, Qiu BY, Su MB, Wu F, Chen DK, Pang T, Gu M, Zhang W, Ma WP, Jiang HW, Li JY, Nan FJ, Li J (2013). Novel small-molecule AMPK activator orally exerts beneficial effects on diabetic db/db mice. Toxicology and applied pharmacology.

[26] Zang M, Xu S, Maitland-Toolan KA, Zuccollo A, Hou X, Jiang B, Wierzbicki M, Verbeuren TJ, Cohen RA (2006). Polyphenols stimulate AMP-activated protein kinase, lower lipids, and inhibit accelerated atherosclerosis in diabetic LDL receptor-deficient mice. Diabetes.

[27] Li J, Qi D, Cheng H, Hu X, Miller EJ, Wu X, Russell KS, Mikush N, Zhang J, Xiao L, Sherwin RS, Young LH (2013). Urocortin 2 autocrine/paracrine and pharmacologic effects to activate AMP-activated protein kinase in the heart. Proceedings of the National Academy of Sciences of the United States of America.

[28] Steinberg GR, Kemp BE (2009). AMPK in Health and Disease. Physiological reviews.

[29] Pavlides S, Tsirigos A, Migneco G, Whitaker-Menezes D, Chiavarina B, Flomenberg N, Frank PG, Casimiro MC, Wang C, Pestell RG, Martinez-Outschoorn UE, Howell A, Sotgia F, Lisanti MP (2010). The autophagic tumor stroma model of cancer: Role of oxidative stress and ketone production in fueling tumor cell metabolism. Cell cycle.

[30] Martinez-Outschoorn UE, Balliet RM, Rivadeneira DB, Chiavarina B, Pavlides S, Wang C, Whitaker-Menezes D, Daumer KM, Lin Z, Witkiewicz AK, Flomenberg N, Howell A, Pestell RG, Knudsen ES, Sotgia F, Lisanti MP (2010). Oxidative stress in cancer associated fibroblasts drives tumor-stroma co-evolution: A new paradigm for understanding tumor metabolism, the field effect and genomic instability in cancer cells. Cell cycle.

[31] Kang J, Shakya A, Tantin D (2009). Stem cells, stress, metabolism and cancer: a drama in two Octs. Trends in biochemical sciences.

[32] William WN, Kim JS, Liu DD, Solis L, Behrens C, Lee JJ, Lippman SM, Kim ES, Hong WK, Wistuba II, Lee HY (2012). The impact of phosphorylated AMP-activated protein kinase expression on lung cancer survival. Annals of oncology: official journal of the European Society for Medical Oncology / ESMO.

[33] Ding L, Getz G, Wheeler DA, Mardis ER, McLellan MD, Cibulskis K, Sougnez C, Greulich H, Muzny DM, Morgan MB, Fulton L, Fulton RS, Zhang Q, Wendl MC, Lawrence MS, Larson DE (2008). Somatic mutations affect key pathways in lung adenocarcinoma. Nature.

[34] Memmott RM, Gills JJ, Hollingshead M, Powers MC, Chen Z, Kemp B, Kozikowski A, Dennis PA (2008). Phosphatidylinositol ether lipid analogues induce AMP-activated protein kinase-dependent death in LKB1-mutant non small cell lung cancer cells. Cancer research.

[35] Carretero J, Medina PP, Blanco R, Smit L, Tang M, Roncador G, Maestre L, Conde E, Lopez-Rios F, Clevers HC, Sanchez-Cespedes M (2007). Dysfunctional AMPK activity, signalling through mTOR and survival in response to energetic stress in LKB1-deficient lung cancer. Oncogene.

[36] Matsumoto S, Iwakawa R, Takahashi K, Kohno T, Nakanishi Y, Matsuno Y, Suzuki K, Nakamoto M, Shimizu E, Minna JD, Yokota J (2007). Prevalence and specificity of LKB1 genetic alterations in lung cancers. Oncogene.

[37] Jemal A, Siegel R, Xu J, Ward E (2010). Cancer statistics, 2010. CA: a cancer journal for clinicians.

[38] Baba Y, Nosho K, Shima K, Meyerhardt JA, Chan AT, Engelman JA, Cantley LC, Loda M, Giovannucci E, Fuchs CS, Ogino S (2010). Prognostic significance of AMP-activated protein kinase expression and modifying effect of MAPK3/1 in colorectal cancer. British journal of cancer.

[39] Petti C, Vegetti C, Molla A, Bersani I, Cleris L, Mustard KJ, Formelli F, Hardie GD, Sensi M, Anichini A (2012). AMPK activators inhibit the proliferation of human melanomas bearing the activated MAPK pathway. Melanoma research.

[40] Zhao Y, Tan Y, Xi S, Li Y, Li C, Cui J, Yan X, Li X, Wang G, Li W, Cai L (2013). A novel mechanism by which SDF-1beta protects cardiac cells from palmitate-induced endoplasmic reticulum stress and apoptosis via CXCR7 and AMPK/p38 MAPK-mediated interleukin-6 generation. Diabetes.

[41] Bosch FX, Ribes J, Diaz M, Cleries R (2004). Primary liver cancer: worldwide incidence and trends. Gastroenterology.

[42] Smith RJ (2013). Nutrition and metabolism in hepatocellular carcinoma. Hepatobiliary surgery and nutrition.

[43] Huang YH, Chen ZK, Huang KT, Li P, He B, Guo X, Zhong JQ, Zhang QY, Shi HQ, Song QT, Yu ZP, Shan YF (2013). Decreased expression of LKB1 correlates with poor prognosis in hepatocellular carcinoma patients undergoing hepatectomy. Asian Pacific journal of cancer prevention: APJCP.

[44] Zheng L, Yang W, Wu F, Wang C, Yu L, Tang L, Qiu B, Li Y, Guo L, Wu M, Feng G, Zou D, Wang H (2013). Prognostic significance of AMPK activation and therapeutic effects of metformin in hepatocellular carcinoma. Clinical cancer research: an official journal of the American Association for Cancer Research.

[45] Cheng J, Huang T, Li Y, Guo Y, Zhu Y, Wang Q, Tan X, Chen W, Zhang Y, Cheng W, Yamamoto T, Jing X, Huang J (2014). AMP-activated protein kinase suppresses the *in vitro* and *in vivo* proliferation of hepatocellular carcinoma. PloS one.

[46] Yi G, He Z, Zhou X, Xian L, Yuan T, Jia X, Hong J, He L, Liu J (2013). Low concentration of metformin induces a p53-dependent senescence in hepatoma cells via activation of the AMPK pathway. International journal of oncology.

[47] Hu M, Huang H, Zhao R, Li P, Li M, Miao H, Chen N, Chen M (2014). AZD8055 induces cell death associated with autophagy and activation of AMPK in hepatocellular carcinoma. Oncology reports.

[48] Kang S, Kim JE, Song NR, Jung SK, Lee MH, Park JS, Yeom MH, Bode AM, Dong Z, Lee KW (2014). The ginsenoside 20-O-beta-D-glucopyranosyl-20(S)-protopanaxadiol induces autophagy and apoptosis in human melanoma via AMPK/JNK phosphorylation. PloS one.

[49] Cerezo M, Tichet M, Abbe P, Ohanna M, Lehraiki A, Rouaud F, Allegra M, Giacchero D, Bahadoran P, Bertolotto C, Tartare-Deckert S, Ballotti R, Rocchi S (2013). Metformin blocks melanoma invasion and metastasis development in AMPK/p53-dependent manner. Molecular cancer therapeutics.

[50] Borgdorff V, Rix U, Winter GE, Gridling M, Muller AC, Breitwieser FP, Wagner C, Colinge J, Bennett KL, Superti-Furga G, Wagner SN (2014). A chemical biology approach identifies AMPK as a modulator of melanoma oncogene MITF. Oncogene.

[51] Ambrosini G, Musi E, Ho AL, de Stanchina E, Schwartz GK (2013). Inhibition of mutant GNAQ signaling in uveal melanoma induces AMPK-dependent autophagic cell death. Molecular cancer therapeutics.

[52] Woodard J, Platanias LC (2010). AMP-activated kinase (AMPK)-generated signals in malignant melanoma cell growth and survival. Biochemical and biophysical research communications.

[53] Taliaferro-Smith L, Nagalingam A, Zhong D, Zhou W, Saxena NK, Sharma D (2009). LKB1 is required for adiponectin-mediated modulation of AMPK-S6K axis and inhibition of migration and invasion of breast cancer cells. Oncogene.

[54] Phoenix KN, Vumbaca F, Claffey KP (2009). Therapeutic metformin/AMPK activation promotes the angiogenic phenotype in the ERalpha negative MDA-MB-435 breast cancer model. Breast cancer research and treatment.

[55] Fox MM, Phoenix KN, Kopsiaftis SG, Claffey KP (2013). AMP-Activated Protein Kinase alpha 2 Isoform Suppression in Primary Breast Cancer Alters AMPK Growth Control and Apoptotic Signaling. Genes & cancer.

[56] Kim HS, Kim MJ, Lim J, Yang Y, Lee MS, Lim JS (2014). NDRG2 overexpression enhances glucose deprivation-mediated apoptosis in breast cancer cells via inhibition of the LKB1-AMPK pathway. Genes & cancer.

[57] Chhipa RR, Wu Y, Ip C (2011). AMPK-mediated autophagy is a survival mechanism in androgen-dependent prostate cancer cells subjected to androgen deprivation and hypoxia. Cellular signalling.

[58] Frigo DE, Howe MK, Wittmann BM, Brunner AM, Cushman I, Wang Q, Brown M, Means AR, McDonnell DP (2011). CaM kinase kinase beta-mediated activation of the growth regulatory kinase AMPK is required for androgen-dependent migration of prostate cancer cells. Cancer research.

[59] Li C, Liu VW, Chiu PM, Yao KM, Ngan HY, Chan DW (2014). Reduced expression of AMPK-beta1 during tumor progression enhances the oncogenic capacity of advanced ovarian cancer. Molecular cancer.

[60] Kandala PK, Srivastava SK (2012). Regulation of macroautophagy in ovarian cancer cells *in vitro* and *in vivo* by controlling glucose regulatory protein 78 and AMPK. Oncotarget.

[61] Vakana E, Platanias LC (2011). AMPK in BCR-ABL expressing leukemias. Regulatory effects and therapeutic implications. Oncotarget.

[62] Hadad SM, Baker L, Quinlan PR, Robertson KE, Bray SE, Thomson G, Kellock D, Jordan LB, Purdie CA, Hardie DG, Fleming S, Thompson AM (2009). Histological evaluation of AMPK signalling in primary breast cancer. BMC cancer.

[63] Zong H, Yin B, Zhou H, Cai D, Ma B, Xiang Y (2014). Inhibition of mTOR pathway attenuates migration and invasion of gallbladder cancer via EMT inhibition. Molecular biology reports.

[64] Inoki K, Zhu T, Guan KL (2003). TSC2 mediates cellular energy response to control cell growth and survival. Cell.

[65] Rubinsztein DC, Gestwicki JE, Murphy LO, Klionsky DJ (2007). Potential therapeutic applications of autophagy. Nature reviews Drug discovery.

[66] Thoreen CC, Kang SA, Chang JW, Liu Q, Zhang J, Gao Y, Reichling LJ, Sim T, Sabatini DM, Gray NS (2009). An ATP-competitive mammalian target of rapamycin inhibitor reveals rapamycin-resistant functions of mTORC1. The Journal of biological chemistry.

[67] Kandadi MR, Hu N, Ren J (2013). ULK1 plays a critical role in AMPK-mediated myocardial autophagy and contractile dysfunction following acute alcohol challenge. Current pharmaceutical design.

[68] Mack HI, Zheng B, Asara JM, Thomas SM (2012). AMPK-dependent phosphorylation of ULK1 regulates ATG9 localization. Autophagy.

[69] Mao K, Klionsky DJ (2011). AMPK activates autophagy by phosphorylating ULK1. Circulation research.

[70] Tripathi DN, Chowdhury R, Trudel LJ, Tee AR, Slack RS, Walker CL, Wogan GN (2013). Reactive nitrogen species regulate autophagy through ATM-AMPK-TSC2-mediated suppression of mTORC1. Proceedings of the National Academy of Sciences of the United States of America.

[71] Prescott SM, Fitzpatrick FA (2000). Cyclooxygenase-2 and carcinogenesis. Biochimica et biophysica acta.

[72] Castellone MD, Teramoto H, Williams BO, Druey KM, Gutkind JS (2005). Prostaglandin E2 promotes colon cancer cell growth through a Gs-axin-beta-catenin signaling axis. Science.

[73] Williams CS, Mann M, DuBois RN (1999). The role of cyclooxygenases in inflammation, cancer, and development. Oncogene.

[74] Williams C, Shattuck-Brandt RL, DuBois RN (1999). The role of COX-2 in intestinal cancer. Annals of the New York Academy of Sciences.

[75] Hwang JT, Kim YM, Surh YJ, Baik HW, Lee SK, Ha J, Park OJ (2006). Selenium regulates cyclooxygenase-2 and extracellular signal-regulated kinase signaling pathways by activating AMP-activated protein kinase in colon cancer cells. Cancer research.

[76] Lee YK, Park SY, Kim YM, Park OJ (2009). Regulatory effect of the AMPK-COX-2 signaling pathway in curcumin-induced apoptosis in HT-29 colon cancer cells. Annals of the New York Academy of Sciences.

[77] Lee JY, Choi AY, Oh YT, Choe W, Yeo EJ, Ha J, Kang I (2012). AMP-activated protein kinase mediates T cell activation-induced expression of FasL and COX-2 via protein kinase C theta-dependent pathway in human Jurkat T leukemia cells. Cellular signalling.

[78] Saini MK, Sharma P, Kaur J, Sanyal SN (2009). The cyclooxygenase-2 inhibitor etoricoxib is a potent chemopreventive agent of colon carcinogenesis in the rat model. Journal of environmental pathology, toxicology and oncology: official organ of the International Society for Environmental Toxicology and Cancer.

[79] Furukawa F, Nishikawa A, Lee IS, Kanki K, Umemura T, Okazaki K, Kawamori T, Wakabayashi K, Hirose M (2003). A cyclooxygenase-2 inhibitor, nimesulide, inhibits postinitiation phase of N-nitrosobis(2-oxopropyl)amine-induced pancreatic carcinogenesis in hamsters. International journal of cancer Journal international du cancer.

[80] Vogelstein B, Lane D, Levine AJ (2000). Surfing the p53 network. Nature.

[81] Vousden KH, Prives C (2009). Blinded by the Light: The Growing Complexity of p53. Cell.

[82] Lee EJ, Kim TJ, Kim DS, Choi CH, Lee JW, Lee JH, Bae DS, Kim BG (2010). p53 alteration independently predicts poor outcomes in patients with endometrial cancer: a clinicopathologic study of 131 cases and literature review. Gynecologic oncology.

[83] Okoshi R, Ozaki T, Yamamoto H, Ando K, Koida N, Ono S, Koda T, Kamijo T, Nakagawara A, Kizaki H (2008). Activation of AMP-activated protein kinase induces p53-dependent apoptotic cell death in response to energetic stress. The Journal of biological chemistry.

[84] Phoenix KN, Devarakonda CV, Fox MM, Stevens LE, Claffey KP (2012). AMPKalpha2 Suppresses Murine Embryonic Fibroblast Transformation and Tumorigenesis. Genes & cancer.

[85] Buzzai M, Jones RG, Amaravadi RK, Lum JJ, DeBerardinis RJ, Zhao F, Viollet B, Thompson CB (2007). Systemic treatment with the antidiabetic drug metformin selectively impairs p53-deficient tumor cell growth. Cancer research.

[86] Zakikhani M, Dowling R, Fantus IG, Sonenberg N, Pollak M (2006). Metformin is an AMP kinase-dependent growth inhibitor for breast cancer cells. Cancer research.

[87] Hardie DG, Pan DA (2002). Regulation of fatty acid synthesis and oxidation by the AMP-activated protein kinase. Biochemical Society transactions.

[88] Yi X, Cao S, Chang B, Zhao D, Gao H, Wan Y, Shi J, Wei W, Guan Y (2013). Effects of acute exercise and chronic exercise on the liver leptin-AMPK-ACC signaling pathway in rats with type 2 diabetes. Journal of diabetes research.

[89] Janovska A, Hatzinikolas G, Staikopoulos V, McInerney J, Mano M, Wittert GA (2008). AMPK and ACC phosphorylation: effect of leptin, muscle fibre type and obesity. Molecular and cellular endocrinology.

[90] Moore F, Brophy PJ (1994). Regulation of acetyl-CoA carboxylase (ACC) by ATP depletion in developing oligodendrocytes mimics the action of AMP-activated protein kinase (AMPK). Biochemical Society transactions.

[91] Feng Z, Hu W, de Stanchina E, Teresky AK, Jin S, Lowe S, Levine AJ (2007). The regulation of AMPK beta1, TSC2, and PTEN expression by p53: stress, cell and tissue specificity, and the role of these gene products in modulating the IGF-1-AKT-mTOR pathways. Cancer research.

[92] Beckers A, Organe S, Timmermans L, Scheys K, Peeters A, Brusselmans K, Verhoeven G, Swinnen JV (2007). Chemical inhibition of acetyl-CoA carboxylase induces growth arrest and cytotoxicity selectively in cancer cells. Cancer research.

[93] O'Reilly KE, Rojo F, She QB, Solit D, Mills GB, Smith D, Lane H, Hofmann F, Hicklin DJ, Ludwig DL, Baselga J, Rosen N (2006). mTOR inhibition induces upstream receptor tyrosine kinase signaling and activates Akt. Cancer research.

[94] Leclerc GM, Leclerc GJ, Fu G, Barredo JC (2010). AMPK-induced activation of Akt by AICAR is mediated by IGF-1R dependent and independent mechanisms in acute lymphoblastic leukemia. Journal of molecular signaling.

[95] Tao R, Gong J, Luo X, Zang M, Guo W, Wen R, Luo Z (2010). AMPK exerts dual regulatory effects on the PI3K pathway. Journal of molecular signaling.

[96] Rattan R, Giri S, Singh AK, Singh I (2005). 5-Aminoimidazole-4-carboxamide-1-beta-D-ribofuranoside inhibits cancer cell proliferation *in vitro* and *in vivo* via AMP-activated protein kinase. The Journal of biological chemistry.

[97] Choudhury Y, Yang Z, Ahmad I, Nixon C, Salt IP, Leung HY (2014). AMP-activated protein kinase (AMPK) as a potential therapeutic target independent of PI3K/Akt signaling in prostate cancer. Oncoscience.

[98] Dandapani M, Hardie DG (2013). AMPK: opposing the metabolic changes in both tumour cells and inflammatory cells?. Biochemical Society transactions.

[99] Carroll KC, Viollet B, Suttles J (2013). AMPKalpha1 deficiency amplifies proinflammatory myeloid APC activity and CD40 signaling. Journal of leukocyte biology.

[100] Hoogendijk AJ, Pinhancos SS, van der Poll T, Wieland CW (2013). AMP-activated protein kinase activation by 5-aminoimidazole-4-carbox-amide-1-beta-D-ribofuranoside (AICAR) reduces lipoteichoic acid-induced lung inflammation. The Journal of biological chemistry.

[101] Zhang Y, Storr SJ, Johnson K, Green AR, Rakha EA, Ellis IO, Morgan DA, Martin SG (2014). Involvement of metformin and AMPK in the radioresponse and prognosis of luminal versus basal-like breast cancer treated with radiotherapy. Oncotarget.

[102] Tebbe C, Chhina J, Dar SA, Sarigiannis K, Giri S, Munkarah AR, Rattan R (2014). Metformin limits the adipocyte tumor-promoting effect on ovarian cancer. Oncotarget.

[103] Morales DR, Morris AD (2014). Metformin in Cancer Treatment and Prevention. Annual review of medicine.

[104] Kasznicki J, Sliwinska A, Drzewoski J (2014). Metformin in cancer prevention and therapy. Annals of translational medicine.

[105] Zhang ZJ, Zheng ZJ, Shi R, Su Q, Jiang Q, Kip KE (2012). Metformin for liver cancer prevention in patients with type 2 diabetes: a systematic review and meta-analysis. The Journal of clinical endocrinology and metabolism.

[106] Yang Y (2011). Metformin for cancer prevention. Frontiers of medicine.

[107] Storozhuk Y, Hopmans SN, Sanli T, Barron C, Tsiani E, Cutz JC, Pond G, Wright J, Singh G, Tsakiridis T (2013). Metformin inhibits growth and enhances radiation response of non-small cell lung cancer (NSCLC) through ATM and AMPK. British journal of cancer.

[108] Memmott RM, Mercado JR, Maier CR, Kawabata S, Fox SD, Dennis PA (2010). Metformin prevents tobacco carcinogen--induced lung tumorigenesis. Cancer prevention research.

[109] Saito T, Chiba T, Yuki K, Zen Y, Oshima M, Koide S, Motoyama T, Ogasawara S, Suzuki E, Ooka Y, Tawada A, Tada M, Kanai F, Takiguchi Y, Iwama A, Yokosuka O (2013). Metformin, a diabetes drug, eliminates tumor-initiating hepatocellular carcinoma cells. PloS one.

[110] Hosono K, Endo H, Takahashi H, Sugiyama M, Uchiyama T, Suzuki K, Nozaki Y, Yoneda K, Fujita K, Yoneda M, Inamori M, Tomatsu A, Chihara T, Shimpo K, Nakagama H, Nakajima A (2010). Metformin suppresses azoxymethane-induced colorectal aberrant crypt foci by activating AMP-activated protein kinase. Molecular carcinogenesis.

[111] Ahren B, Mathieu C, Bader G, Schweizer A, Foley JE (2014). Efficacy of vildagliptin versus sulfonylureas as add-on therapy to metformin: comparison of results from randomised controlled and observational studies. Diabetologia.

[112] Mantovani A, Allavena P, Sica A, Balkwill F (2008). Cancer-related inflammation. Nature.

[113] Din FV, Theodoratou E, Farrington SM, Tenesa A, Barnetson RA, Cetnarskyj R, Stark L, Porteous ME, Campbell H, Dunlop MG (2010). Effect of aspirin and NSAIDs on risk and survival from colorectal cancer. Gut.

[114] Rothwell PM, Wilson M, Elwin CE, Norrving B, Algra A, Warlow CP, Meade TW (2010). Long-term effect of aspirin on colorectal cancer incidence and mortality: 20-year follow-up of five randomised trials. Lancet.

[115] Grossman HB (2003). Selective COX-2 inhibitors as chemopreventive and therapeutic agents. Drugs of today.

[116] Din FV, Valanciute A, Houde VP, Zibrova D, Green KA, Sakamoto K, Alessi DR, Dunlop MG (2012). Aspirin inhibits mTOR signaling, activates AMP-activated protein kinase, and induces autophagy in colorectal cancer cells. Gastroenterology.

[117] Russe OQ, Moser CV, Kynast KL, King TS, Stephan H, Geisslinger G, Niederberger E (2013). Activation of the AMP-activated protein kinase reduces inflammatory nociception. The journal of pain: official journal of the American Pain Society.

[118] Mann J (2002). Natural products in cancer chemotherapy: past, present and future. Nature reviews Cancer.

[119] Lee DH, Lee TH, Jung CH, Kim YH (2012). Wogonin induces apoptosis by activating the AMPK and p53 signaling pathways in human glioblastoma cells. Cellular signalling.

[120] Yun SM, Jung JH, Jeong SJ, Sohn EJ, Kim B, Kim SH (2014). Tanshinone IIA induces autophagic cell death via activation of AMPK and ERK and inhibition of mTOR and p70 S6K in KBM-5 leukemia cells. Phytotherapy research: PTR.

[121] Russo GL (2007). Ins and outs of dietary phytochemicals in cancer chemoprevention. Biochemical pharmacology.

[122] Lee YK, Park SY, Kim YM, Lee WS, Park OJ (2009). AMP kinase/cyclooxygenase-2 pathway regulates proliferation and apoptosis of cancer cells treated with quercetin. Experimental & molecular medicine.

[123] Park IJ, Yang WK, Nam SH, Hong J, Yang KR, Kim J, Kim SS, Choe W, Kang I, Ha J (2014). Cryptotanshinone induces G1 cell cycle arrest and autophagic cell death by activating the AMP-activated protein kinase signal pathway in HepG2 hepatoma. Apoptosis: an international journal on programmed cell death.

[124] Puissant A, Robert G, Fenouille N, Luciano F, Cassuto JP, Raynaud S, Auberger P (2010). Resveratrol promotes autophagic cell death in chronic myelogenous leukemia cells via JNK-mediated p62/SQSTM1 expression and AMPK activation. Cancer research.

[125] Park JB, Lee MS, Cha EY, Lee JS, Sul JY, Song IS, Kim JY (2012). Magnolol-induced apoptosis in HCT-116 colon cancer cells is associated with the AMP-activated protein kinase signaling pathway. Biological & pharmaceutical bulletin.

[126] Hwang JT, Ha J, Park IJ, Lee SK, Baik HW, Kim YM, Park OJ (2007). Apoptotic effect of EGCG in HT-29 colon cancer cells via AMPK signal pathway. Cancer letters.

[127] Kang MR, Park SK, Lee CW, Cho IJ, Jo YN, Yang JW, Kim JA, Yun J, Lee KH, Kwon HJ, Kim BW, Lee K, Kang JS, Kim HM (2012). Widdrol induces apoptosis via activation of AMP-activated protein kinase in colon cancer cells. Oncology reports.

[128] Zhang Y, Xu S, Lin J, Yao G, Han Z, Liang B, Zou Z, Chen Z, Song Q, Dai Y, Gao T, Liu A, Bai X (2012). mTORC1 is a target of nordihydroguaiaretic acid to prevent breast tumor growth *in vitro* and *in vivo*. Breast cancer research and treatment.

[129] Shieh JM, Chen YC, Lin YC, Lin JN, Chen WC, Chen YY, Ho CT, Way TD (2013). Demethoxycurcumin inhibits energy metabolic and oncogenic signaling pathways through AMPK activation in triple-negative breast cancer cells. Journal of agricultural and food chemistry.

[130] Pan W, Yang H, Cao C, Song X, Wallin B, Kivlin R, Lu S, Hu G, Di W, Wan Y (2008). AMPK mediates curcumin-induced cell death in CaOV3 ovarian cancer cells. Oncology reports.

[131] Song G, Mao YB, Cai QF, Yao LM, Ouyang GL, Bao SD (2005). Curcumin induces human HT-29 colon adenocarcinoma cell apoptosis by activating p53 and regulating apoptosis-related protein expression. Brazilian journal of medical and biological research = Revista brasileira de pesquisas medicas e biologicas / Sociedade Brasileira de Biofisica [et al].

[132] Khanal P, Oh WK, Yun HJ, Namgoong GM, Ahn SG, Kwon SM, Choi HK, Choi HS (2011). p-HPEA-EDA, a phenolic compound of virgin olive oil, activates AMP-activated protein kinase to inhibit carcinogenesis. Carcinogenesis.

[133] Chiang PC, Lin SC, Pan SL, Kuo CH, Tsai IL, Kuo MT, Wen WC, Chen P, Guh JH (2010). Antroquinonol displays anticancer potential against human hepatocellular carcinoma cells: a crucial role of AMPK and mTOR pathways. Biochemical pharmacology.

[134] Nagalingam A, Arbiser JL, Bonner MY, Saxena NK, Sharma D (2012). Honokiol activates AMP-activated protein kinase in breast cancer cells via an LKB1-dependent pathway and inhibits breast carcinogenesis. Breast cancer research: BCR.

[135] Li W, Hua B, Saud SM, Lin H, Hou W, Matter MS, Jia L, Colburn NH, Young MR (2014). Berberine regulates AMP-activated protein kinase signaling pathways and inhibits colon tumorigenesis in mice. Molecular carcinogenesis.

[136] Santidrian AF, Gonzalez-Girones DM, Iglesias-Serret D, Coll-Mulet L, Cosialls AM, de Frias M, Campas C, Gonzalez-Barca E, Alonso E, Labi V, Viollet B, Benito A, Pons G, Villunger A, Gil J (2010). AICAR induces apoptosis independently of AMPK and p53 through up-regulation of the BH3-only proteins BIM and NOXA in chronic lymphocytic leukemia cells. Blood.

[137] Sengupta TK, Leclerc GM, Hsieh-Kinser TT, Leclerc GJ, Singh I, Barredo JC (2007). Cytotoxic effect of 5-aminoimidazole-4-carboxamide-1-beta-4-ribofuranoside (AICAR) on childhood acute lymphoblastic leukemia (ALL) cells: implication for targeted therapy. Molecular cancer.

[138] Su RY, Chao Y, Chen TY, Huang DY, Lin WW (2007). 5-Aminoimidazole-4-carboxamide riboside sensitizes TRAIL- and TNF{alpha}-induced cytotoxicity in colon cancer cells through AMP-activated protein kinase signaling. Molecular cancer therapeutics.

[139] Wang S, Penchala S, Prabhu S, Wang J, Huang Y (2010). Molecular basis of traditional Chinese medicine in cancer chemoprevention. Current drug discovery technologies.

[140] Yin J, Zhang H, Ye J (2008). Traditional chinese medicine in treatment of metabolic syndrome. Endocrine, metabolic & immune disorders drug targets.

[141] Huang Z, Xu X, Lu F, Wang N, Chen G, Zhao Y, Zou X, Wang K, Dong H, Xu L (2013). Jiao tai wan attenuates hepatic lipid accumulation in type 2 diabetes mellitus. Evidence-based complementary and alternative medicine: eCAM.

[142] Yin J, Luo Y, Deng H, Qin S, Tang W, Zeng L, Zhou B (2014). Hugan Qingzhi medication ameliorates hepatic steatosis by activating AMPK and PPARalpha pathways in L02 cells and HepG2 cells. Journal of ethnopharmacology.

[143] Rios M, Foretz M, Viollet B, Prieto A, Fraga M, Costoya JA, Senaris R (2013). AMPK activation by oncogenesis is required to maintain cancer cell proliferation in astrocytic tumors. Cancer research.

[144] Martin MJ, Hayward R, Viros A, Marais R (2012). Metformin accelerates the growth of BRAF V600E-driven melanoma by upregulating VEGF-A. Cancer discovery.

[145] Rokhlin OW, Taghiyev AF, Bayer KU, Bumcrot D, Koteliansk VE, Glover RA, Cohen MB (2007). Calcium/calmodulin-dependent kinase II plays an important role in prostate cancer cell survival. Cancer biology & therapy.

